# Assessment of Blood-Count-Derived Biomarkers, Homocysteine Levels, MTHFR Mutation, and Clinical Manifestations in Severe Peripheral Artery Disease

**DOI:** 10.3390/biomedicines14010210

**Published:** 2026-01-18

**Authors:** Orsolya-Zsuzsa Akácsos-Szász, Zsuzsánna Simon-Szabó, Ana-Claudia Cârstea, Liliana Demian, Róbert Nemes-Nagy, Sándor Pál, Raluca-Maria Tilinca, Mónika Szilveszter, Adrian Man, Mariana Cornelia Tilinca, Enikő Nemes-Nagy

**Affiliations:** 1 Doctoral School, George Emil Palade University of Medicine, Pharmacy, Science, and Technology of Targu Mures, 540142 Targu Mures, Romania; 2 Department of Surgery, Gheorgheni City Hospital, 535500 Gheorgheni, Romania; 3 Department of Pathophysiology, George Emil Palade University of Medicine, Pharmacy, Science, and Technology of Targu Mures, 540142 Targu Mures, Romania; 4 Department of Neonatology, Emergency County Hospital of Târgu Mureș, 540142 Targu Mures, Romania; 5 Department of Molecular Biology, Center for Advanced Medical and Pharmaceutical Research, George Emil Palade University of Medicine, Pharmacy, Science, and Technology of Targu Mures, 540142 Targu Mures, Romania; 6 Department of Immunology, Center for Advanced Medical and Pharmaceutical Research, George Emil Palade University of Medicine, Pharmacy, Science, and Technology of Targu Mures, 540142 Targu Mures, Romania; 7 Central Laboratory, Emergency County Hospital of Târgu Mureș, 540136 Targu Mures, Romania; eniko.nemes-nagy@umfst.ro; 8 Faculty of Medicine, George Emil Palade University of Medicine, Pharmacy, Science, and Technology of Targu Mures, 540142 Targu Mures, Romania; 9 Department of Transfusion Medicine, Department of Laboratory Medicine, Medical School, University of Pécs, 7624 Pecs, Hungary; 10 Department of Plastic Surgery, Emergency County Hospital of Târgu Mureș, 540136 Targu Mures, Romania; 11 Department of Microbiology, George Emil Palade University of Medicine, Pharmacy, Science, and Technology of Targu Mures, 540136 Targu Mures, Romania; 12 Department of Medical Laboratory, County Hospital of Târgu Mureș, 540136 Targu Mures, Romania; 13 Department of Internal Medicine I, George Emil Palade University of Medicine, Pharmacy, Science, and Technology of Targu Mures, 540136 Targu Mures, Romania; 14 Department of Diabetes, Nutrition and Metabolic Diseases, Emergency County Hospital of Târgu Mureș, 540136 Targu Mures, Romania; 15 Department of Chemistry and Medical Biochemistry, George Emil Palade University of Medicine, Pharmacy, Science, and Technology of Targu Mures, 540136 Targu Mures, Romania

**Keywords:** blood-count-derived biomarkers, diabetes mellitus, homocysteine, methylenetetrahydrofolate reductase mutation, peripheral artery disease

## Abstract

**Background:** Infection and consequent limb amputations are complications of severe peripheral artery disease, especially in diabetic patients. Risk factors and prognostic markers are of particular importance in defining patient care. **Methods**: This study included 99 peripheral artery disease (PAD) patients admitted for surgical intervention in the 2020–2021 time interval. The included subjects were stratified by type 2 diabetes mellitus (T2DM) diagnosis (present/absent). Protein, albumin concentrations, blood-count-derived inflammatory markers, and cultures from gangrenous wounds were assessed. In the group of severe cases, needing lower limb amputation (*n* = 31), homocysteine level, and related methylene tetrahydrofolate reductase (MTHFR) gene mutations were also investigated. **Results:** The mean age of patients was 68.36 ± 11.79 (SD) years and T2DM patients were significantly older (*p* = 0.0303). The measured inflammatory markers were at normal values in 20% of the subjects. In the cohort of infected patients, *S. aureus*, *P. mirabilis*, *P. vulgaris*, and *S. agalactiae* were the most commonly identified bacteria, with *C. albicans* prevailing as the most common fungal pathogen. The patient length of stay (LoS) was significantly longer in patients with pathological blood-count-derived biomarkers (*p* = 0.0283). A total of 58% of the severe cases presented hyperhomocysteinemia (mean 17.7 ± 10.6 (SD) μmol/L), and 19% of them presented homozygous mutation of the MTHFR gene (C677T), while 39% carried a heterozygous mutation. Compared to those with normal alleles, homocysteine levels were significantly higher in subjects with homozygous mutation (*p* = 0.0334). **Discussion:** Homozygous MTHFR mutation was associated with hyperhomocysteinemia. Blood-count-derived inflammatory markers may indicate an unfavorable outcome for PAD patients, guiding clinicians in identifying patients that are prone to complications.

## 1. Introduction

Peripheral artery disease (PAD) is a pathological condition with an increasing prevalence worldwide, particularly among the elderly, tobacco users, and patients with diabetes mellitus. In most severe cases, it leads to limb amputation [1].

Abnormal vascular tone and dysregulated coagulation on an atherosclerotic background promote the development of peripheral arterial thrombosis [2]. Inflammation and enhanced thrombin production in the vessels contribute to the development of arteriopathy in the lower limbs [3]. Elevated levels of specific proinflammatory cytokines, such as interleukin 6, are useful predictive factors for poor outcomes and prolonged hospital stays in patients with arteriopathy [4]. A high C-reactive protein (CRP) to albumin ratio is associated with increased mortality and limb amputation risk in patients with PAD [5]. Albumin has anti-inflammatory, antioxidant, and antithrombotic properties and is a marker of nutritional status. Pre-operative hypoalbuminemia is associated with poor postoperative outcomes following procedures for lower limb arterial disease [6].

Genetic predisposition to thrombosis or atherosclerosis, especially in diabetic patients, may trigger PAD, which occurs twice as frequently in diabetic patients than in the general population. Moreover, PAD is a risk for diabetic foot ulcers, contributing to several complications [7,8]. The most common mutation of MTHFR (C677T) leads to a decrease in enzyme activity following the mis-incorporated amino acid, valine instead of alanine, at position 222, and impairing the re-methylation of homocysteine (Hcy) to methionine. The above-mentioned mutation can be found in hereditary disorders that predispose individuals to thrombosis, which is why it is included in thrombophilia screening tests. Hyperhomocysteinemia is an important risk factor for the development of thrombosis [9,10]. Hcy enhances vasculopathy by directly damaging the endothelium, which reduces plasma nitric oxide (NO) levels, thus inhibiting NO-mediated vasodilation and causing hypoperfusion [11]. This sulfur-containing amino acid induces oxidative stress and inflammation in the endothelium, thus promoting atherosclerosis and enhanced oxidation of low-density lipoproteins, which leads to narrowing and stiffness of blood vessels, restricted blood flow, and plaque instability [12,13].

Several derived parameters can be generated using the values of the complete blood count (CBC) for assessment of inflammatory state, enhancing postoperative prognosis. Neutrophil-to-lymphocyte ratio (NLR), platelet-to-lymphocyte ratio (PLR), lymphocyte-to-monocyte ratio (LMR) and platelet count/mean platelet volume ratio (PC/MPV = MPMV) are well-known indicators of systemic inflammation [14,15], while platelet-to-monocyte ratio (PMR) is less commonly used as an inflammatory parameter. Elevated NLR levels, as an inflammatory marker, correlate more strongly with cardiovascular risk than elevated CRP levels in patients with T2DM. An elevated NLR level is a poor prognostic biomarker in cardiovascular diseases, including PAD, in both diabetic and non-diabetic patients [16]. An increased platelet count and decreased MPV are associated with infections or inflammatory response; their ratio represents a promising prognostic factor in infectious conditions (high values indicate poor prognosis) [14].

The aim of this study was to investigate albuminemia, proteinemia, and blood-count-derived biomarkers as potential predictors of outcomes in hospitalized diabetic and non-diabetic PAD patients who underwent surgery during the COVID-19 pandemic. The prevalence of pathogens present in cases that were complicated by infections was also defined. A genetic test for predisposition to thrombosis (MTHFR mutation) and serum homocysteine levels were measured in selected subjects with severe lower limb arterial disease who were willing to participate, using blood samples collected prior to surgery.

## 2. Materials and Methods

This cross-sectional study was conducted between 2020 and 2021 and evaluated 99 diabetic and non-diabetic patients admitted to the General Surgery Department of the County Clinical Hospital in Târgu-Mureș for surgical intervention of the lower limb, amputation, or necrectomy. Patients with documented peripheral artery disease requiring surgery were included, while pregnancy and trauma-related vasculopathy represented exclusion from the study. The PAD diagnosis was established based on clinical examination (including observation of the limb, pulse palpation) and confirmed by Doppler ultrasound and angiography. Rutherford and Fontaine classifications were used to classify the clinical stage of PAD. The cohort was stratified by type 2 diabetes mellitus into diabetic and non-diabetic subgroups.

Based on the Rutherford and Fontaine scales, further stratification was determined for affected patients who met the criteria for critical PAD (grade IV, with ulcers and gangrene). A total of 31 critical PAD cases were referred for further investigation prior to surgery: serum homocysteine measurement and genetic testing for MTHFR mutation. Day-surgery procedures and cases involving only necrectomy were excluded from the subgroup undergoing more extensive testing. The study was approved by the Ethics Committee of the hospital and the George Emil Palade University of Medicine, Pharmacy, Science, and Technology of Targu Mures (approval nr. 753/26.02.2020). The study was conducted in accordance with the Declaration of Helsinki.

Venous blood samples for serum protein, albumin, and homocysteine assessment were collected in tubes containing a clot separator. After, centrifugation serum samples were used for routine biochemistry analysis (including protein and albumin measurement) in the hospital’s laboratory. Serum protein and albumin levels were measured by photometric methods, using Architect c4000 equipment (Abbott Laboratories, Abbott Park, IL, USA). Samples for coagulation tests were collected in citrate-containing vacutainers; those for hematological and genetics tests were whole blood samples on EDTA as an anticoagulant. Complete blood count was measured by using a Cell Dyn Ruby hematology analyzer from Abbott Laboratories (Abbott Laboratories, Abbott Park, IL, USA), using consumables from the same manufacturer. Sysmex CS 2500 equipment (Sysmex Corporation, Kobe, Japan) was used for coagulation tests to determine prothrombin time (PT) and activated partial thromboplastin time (APTT). Serum and whole blood samples were stored frozen at −80 °C for further processing at the Medical and Pharmaceutical Advanced Research Center (MPhARC) in Târgu Mureș.

Measurement of the serum homocysteine level was performed in 2022 for the most severe cases by photometric method at the MPhARC on a Cobas Integra 400 Plus analyzer (Roche Diagnostics Ltd., Rotkreuz, Switzerland), using a kit purchased from the same manufacturer.

Whole blood was collected in EDTA tubes (50 mmol/L). Genomic DNA was extracted using the Quick-DNA Miniprep Plus Kit (Zymo Research, Irvine, CA, USA). Genotyping was performed on a 7500 Real-Time PCR System (Applied Biosystems, Thermo Fisher Scientific, Waltham, MA, USA), using TaqMan^®^ SNP Genotyping Assays (Thermo Fisher Scientific, USA) in the Molecular Biology Unit of MPhARC to identify the most common mutation of the MTHFR gene (C677T, Ala222Val), according to the manufacturer’s protocol. The analyzed SNP was rs1801133 (Assay ID: C___1202883_20; Thermo Fisher Scientific, USA).

Pus samples from lower limb wounds of gangrene patients were cultured using classical microbiological methods to isolate the bacteria and fungi, and a microbiological smear was examined using Gram staining. A COVID-19 test was performed using Quantstudio5 Real Time PCR equipment (Thermo Fisher Scientific, USA) and GeneFinder COVID-19 Plus RealAmp IVD kit (Osang Healthcare Co. Ltd., Anyang-si gyeongg, Republic of Korea). Prior to laboratory testing, an individual identification number was assigned to each sample.

### Statistical Analysis

Statistical analyses were performed using the R statistical software environment (version 4.5.1; Vienna, Austria) and GraphPad InStat version 3. Data management and manipulation were conducted using the ‘tidyverse’ (v 2.0.0.) suite of packages: specifically, ’readxl’ (v 1.4.5.) for data import and ‘dplyr’ (v 1.1.4) for data wrangling. To facilitate analysis, column names were programmatically standardized for consistency. Several derived hematological biomarkers were calculated, including the neutrophil-to-lymphocyte ratio (NLR), platelet-to-lymphocyte ratio (PLR), lymphocyte-to-monocyte ratio (LMR), platelet-to-monocyte ratio (PMR), and platelet-to-mean platelet volume ratio (PMPV). Additionally, categorical variables were engineered to define study subgroups, including infection status (infect/non-infect), diabetes status (diabetic/non-diabetic), and mutation status (categorized as homozygous, heterozygous, or normal).

A comprehensive descriptive analysis was performed to characterize the study cohort. The ‘skimr’ package (v 2.1.5.) was used for the initial data quality assessment. Detailed descriptive statistics—including mean, standard deviation (SD), median, interquartile range (IQR), minimum, and maximum—were calculated for all numeric variables. These summaries were stratified by diabetes status, infection status, and the subset patient data by MTHFR mutation status. Publication-quality descriptive tables were generated using the ‘gt’ package (1.2.0).

To identify significant differences in numeric variables between binary groups, a systematic, automated hypothesis-testing procedure was implemented in R. The choice of statistical test was determined by the data distribution. The Shapiro–Wilk test (shapiro.test) was applied to each subgroup to test for normality. If data in both subgroups followed a normal distribution (*p* > 0.05), the parametric unpaired Student’s *t*-test (t.test) was performed. If data in at least one subgroup violated the normality assumption, the non-parametric Wilcoxon rank-sum test (wilcox.test) was utilized. This test is more robust and shows higher precision in handling data with non-normal distribution. In specific instances, GraphPad InStat3 was used for unpaired Student *t*-tests or Mann–Whitney tests to verify comparisons.

The diagnostic utility of the hematological ratios (NLR, PLR, LMR, PMR, and PMPV) was evaluated using receiver operating characteristic (ROC) curve analysis via the pROC package (v 1.19.0.1). Analyses were conducted to predict clinical outcomes (infection, diabetes) and genetic profiles (homozygous/heterozygous mutations). For each parameter, the area under the curve (AUC), sensitivity, and specificity were calculated. The optimal cut-off values were determined using Youden’s J statistics. After performing the ROC analysis, the results were visualized by extracting statistical data, which are presented in tables and comparative box plots to illustrate the differences between the groups.

To investigate the relationships between inflammatory markers and clinical outcomes, such as length of stay, correlation analyses were performed. While Pearson correlation was utilized for preliminary checks in GraphPad InStat3, the final correlation matrix was generated in R, using Spearman’s rank correlation coefficient (cor.test with method = “spearman”) to account for the non-normal distribution of the hematological variables. The results were visualized using a correlogram generated with ‘ggplot2’ (v 3.5.2) and ‘cowplot’ (v 1.1.3) libraries, displaying histograms on the diagonal, scatter plots with regression lines in the upper triangle, and correlation coefficients (rho) with significance levels in the lower triangle. All statistical tests were two-sided, and a *p*-value < 0.05 was considered statistically significant.

## 3. Results

The cohort had a mean age of 68.36 ± 11.79 (SD) years, and male sex was predominant (78 males and 21 females). The cohort was split between rural (58 patients) and urban (41 patients) environment. Within the cohort, 51 patients were classified as diabetic and 48 as non-diabetic. Diabetic subjects were significantly older compared to the non-diabetic ones (*p* = 0.0303). In the cohort, 35 cases presented with infectious gangrene and 15 of the 35 patients had T2DM.

Overall, the average LoS was 10.81 ± 7.44 (SD) days (median: 9 days). The LoS of diabetic (10.60 ± 6.35 (SD) days) and non-diabetic (11.04 ± 8.51 (SD) days) subjects did not differ significantly (*p* = 0.7425).

Pre-operative hypoalbuminemia was present in 75% of the patients, while hypoproteinemia occurred in 25% of the patients before surgical intervention (Table 1). No correlation could be found between the LoS and serum albumin (r = −0.01551, *p* = 0.9618) or protein (r = −0.2028, *p* = 0.1577) concentrations.

Efficient anticoagulation was observed in 7.3% of the patients, based on the PT and APTT values (PT: international normalized ratio (INR) exceeding 2 and APTT exceeding 65 s); one of them had a critically high INR value (8.14) that was attributable to the use of a coumarin derivative.

**Figure 1:**
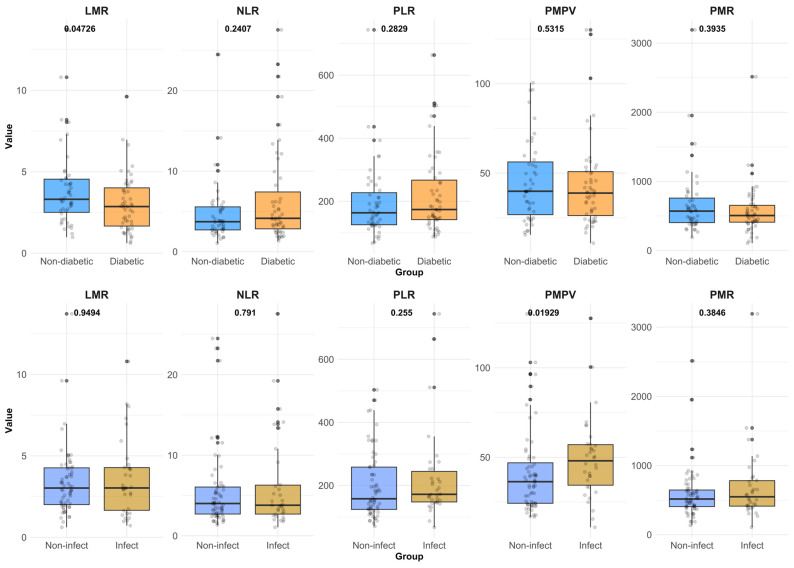
Values of CBC-derived biomarkers in diabetic vs. non-diabetic PAD patients and in subjects with and without infectious gangrene.

The mean NLR was 5.71 ± 11.79 (SD), ranging between 0.52 and 6.75. NLR exceeded 3 (considered the upper limit of normal) in 69% of the patients. A significantly higher mean NLR was found in patients from an urban environment, 6.96 ± 6.32 (SD), compared to those from rural areas, 4.85 ± 4.04 (SD) (*p* = 0.0497). Slightly higher values were described in patients with infected lower limb gangrene, 6.30 ± 5.93 (SD), compared to those without this pathology, 5.40 ± 4.74 (SD), although the difference was not significant (*p* = 0.4143). NLR was slightly higher in diabetic PAD subjects (6.58 ± 6.02 (SD)) compared to non-diabetic PAD patients (5.03 ± 4.35 (SD)) (*p* = 0.2883). Mean PLR was 207.81 ± 121.02 (SD) in the study group of patients with PAD (min: 67.33 and max 743.69). The difference between the mean PLR in patients with T2DM, 219.60 ± 124.95 (SD), was not significant (*p* = 0.2978) compared to non-diabetic subjects, 195.83 ± 115.41 (SD). Mean LMR was 3.46 ± 2.17 (SD), ranging between 0.62 and 13.71. The mean LMR value obtained in diabetic subjects, 3.03 ± 1.76 (SD), versus non-diabetic patients, 3.86 ± 2.44 (SD), showed a significant difference (*p* = 0.0473) (Figure 1).

Diabetic PAD patients (*n* = 51) had significantly lower (*p* = 0.0174) mean hemoglobin (Hb) values, 11.66 ± 2.16 (SD) g/dL, compared to their non-diabetic peers (*n* = 48), 12.57 ± 2.52 (SD) g/dL, and significantly higher (*p* < 0.001) blood glucose values were obtained in diabetic subjects, 172.52 ± 90.15 mg/dL, (SD), compared to the non-diabetic participants, 117.24 ± 54 mg/dL (SD).

**Figure 2:**
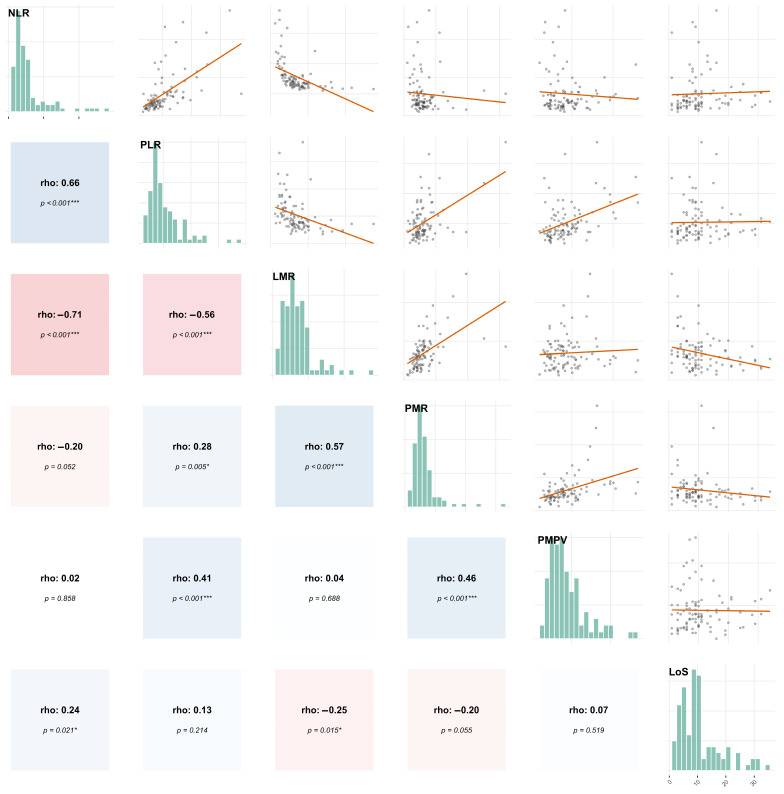
Correlations (Spearman’s rho) between derived inflammatory markers in patients with PAD. The main axis of the correlogram exhibits the data distribution. Positive correlation is represented in red, while negative correlation is in blue. Linear fit is represented by the orange line on the scatter-diagrams. Significant *p*-values were marked using *. The color intensity shows the strength of the correlation.

A positive correlation was found between NLR and PLR, whereas LMR exhibited a negative correlation with both NLR and PLR (Figure 2). The correlations were performed based on the assumption of non-normal distribution of the data (as shown in Figure 2).

The LoS correlated negatively with LMR values. LoS was significantly shorter (*p* = 0.0283) in patients showing normal-range blood-count-derived inflammatory biomarker values compared to those with at least one pathological biomarker (7.55 ± 6.94 (SD) vs. 11.60 ± 7.44 (SD) days).

The mean value of the PC/MPV ratio (PMPV) was significantly higher (*p* = 0.01929) in PAD patients with infectious gangrene, 49.23 ± 23.09 (SD), compared to those without this complication, 41.59 ± 23.79 (SD) (Figure 1). A highly significant difference (*p* < 0.001) was present between the mean MPV values of these two subgroups: 7.63 ± 1.27 (SD) in the infected group compared to 8.82 ± 1.67 (SD) in those without infectious gangrene.

ROC (receiver operating characteristic) analysis defined the predictive accuracy of these parameters and identified the optimal cut-off values for different diagnostic settings. The table below (Table 2) shows the diagnostic utility of the derived hematological ratios in predicting the outcome of certain clinical conditions, including infectious status, diabetes, and the presence of genetic mutations (overall, homozygous, and heterozygous states).

The most common fungal pathogen was *Candida albicans*, which was present in four patients, and *Candida parapsilosis* in one case. The most common bacteria isolated from pus was *Staphylococcus aureus*. The distribution of pathogens found in the pus of PAD patients with infectious gangrene is presented in Table 3.

The antibiotic resistance of the majority of the Gram-negative bacteria isolated from pus samples (*n* = 31) extended to both ampicillin and amoxicillin–clavulanic acid. Specifically, 58.06% of the Gram-negative isolates were resistant to ampicillin (*n* = 18), while 41.94% showed resistance to amoxicillin–clavulanic acid (*n* = 13). Nearly half of the Gram-negative isolates (48.39%; *n* = 15) were resistant to cephalosporins: most commonly, to cefuroxime, a second-generation cephalosporin. Resistance to fluoroquinolones was less prevalent, occurring in only 16.13% of isolates (*n* = 5). Overall, 38.71% of Gram-negative bacteria isolated from gangrenous pus samples showed multidrug resistance.

With regard to Gram-positive bacteria isolated from pus, 40% of *Enterococcus* strains (two from five strains) were resistant to gentamicin and streptomycin. In addition to very frequent resistance to benzylpenicillin (present in 75% of the cases, *n* = 9), half of the *Staphylococcus aureus* strains were simultaneously resistant to tetracycline (*n* = 6); macrolides (erythromycin); and lincosamides (clindamycin) in 41.67% (*n* = 5).

A positive COVID-19 test was obtained in three cases (3%) of the PAD patients during hospitalization for lower limb surgery; one of them also had T2DM.

**Figure 3:**
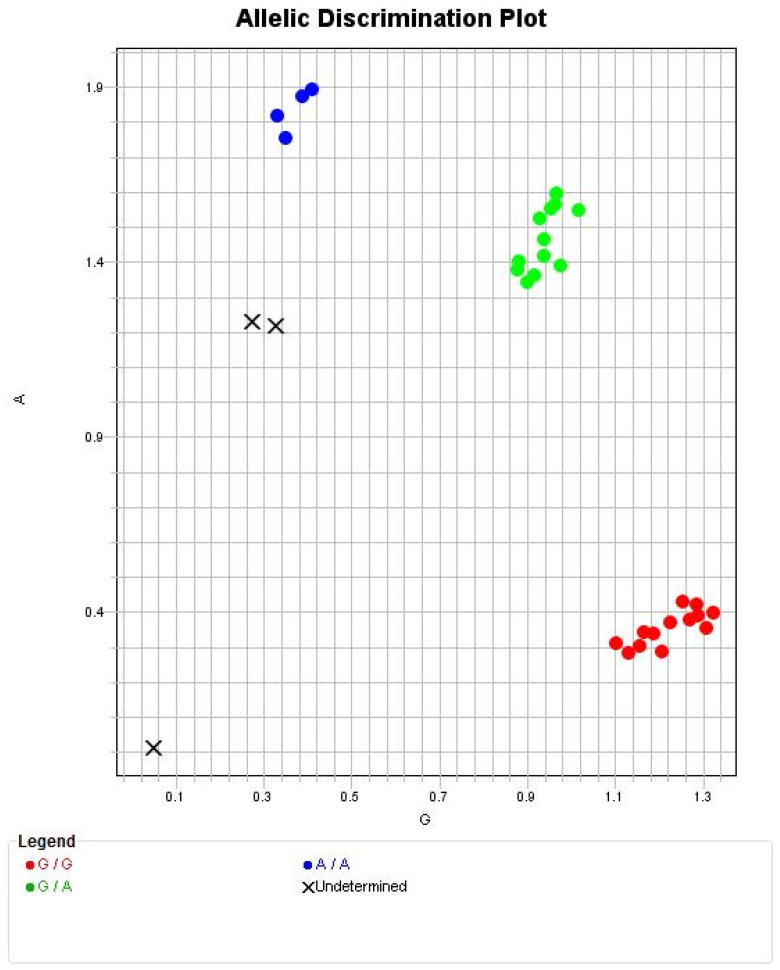
Allelic distribution of the selected critically severe PAD patients. Upper undetermined samples (×) were included in the A/A group. Explanation of the alleles—G/G; G/A; and A/A.

A total of 31 critically severe PAD cases were selected for additional investigations (homocysteine levels and MTHFR genetic mutation), based on their Rutherford and Fontaine score of 4. In the above-mentioned subgroup, patients’ fingers, feet, or lower limb were amputated. In the subgroup assessed for genetic mutation, the distribution of the MTHFR genetic status was 12 heterozygous (G/A), 6 homozygous (A/A), and 13 without mutation (G/G). Homozygous mutation of the MTHFR gene (C677T, Ala222Val) was observed in 19% of the selected patients, heterozygous mutation occurred in 39% of these subjects (Figure 3), 28% were females, and the mean age was 71.1 ± 12.9 (SD) years. T2DM occurred in about two thirds of the patients in this subgroup. Homocysteinemia was only in the normal range (under 12 μmol/L) in 42% of these selected subjects; the mean concentration was 17.7 ± 10.6 (SD) μmol/L, ranging between 3.96 and 44.38 μmol/L.

Significantly higher (*p* = 0.0334) serum homocysteine levels were measured in patients with homozygous mutation (29.61 ± 14.44 (SD) μmol/L) compared to patients without gene mutations (16.23 ± 7.73 (SD) μmol/L), but there was no significant difference (*p* > 0.05) when compared to heterozygous MTHFR mutation homocysteine values (12.54 ± 5.52 (SD) μmol/L). No correlation was found between homocysteniemia and length of hospital stay (r = −0.1695, *p* = 0.4284).

## 4. Discussion

The study found no difference between the examined laboratory parameters and the blood-derived biomarkers among diabetic and non-diabetic subjects, even though diabetes mellitus is one of the most important risk factors for PAD, except for the significantly higher mean blood glucose (as expected) and lower Hb and LMR values in subjects with diabetes. The higher occurrence of anemia in diabetes is similar to that in the literature data, and it is partially due to impaired renal function. Lower Hb values represent an independent risk factor for higher rates of amputation and mortality in patients with diabetic foot ulcers [17]. The considerably older age of T2DM patients in the studied group compared to the non-diabetic group is presumably due to the fact that diabetics are more informed about the conditions associated with their disease, adhere more closely to medical recommendations, and are more attentive to their treatment and lower limb hygiene. This may explain why amputation was only necessary later in life, unlike their non-diabetic counterparts with PAD. Similar age differences have been reported in other studies, such as a recent study evaluating macro- and microvascular dysfunction in T2DM and non-diabetic subjects, which found that diabetic subjects were significantly older than their non-diabetic counterparts [18].

As described in the literature, blood-count-derived biomarkers (NLR, PLR, LMR, PC/MPV, and PMR) are readily accessible, inexpensive and useful parameters in patients with peripheral arteriopathy, especially in cases that are complicated with infections [14,19]. These parameters can be applied on a large scale without high costs in areas with low-income populations [20]. In this study, only LMR values showed an inverse correlation with the length of hospital stay among the derived markers, but the overall hospital stay was significantly shorter for patients with normal values for all derived parameters. The findings of the study provide support for the potential prognostic role of these markers, particularly in cases with complications. Based on the results of the study, it would be advisable to use all these markers together in patients with diabetic and non-diabetic PAD, as this would allow for a more accurate risk assessment. Unfortunately, most of the patients are in an advanced phase of PAD when they are admitted to the General or Vascular Surgery Department. PMR is novel, promising a derived inflammatory parameter which can be used as a prognostic marker in different diseases, including cardiovascular pathology. The increased number of monocytes indicates a persistent inflammatory response, which can lead to low PMR and poor prognostic outcomes [21].

The significantly higher NLR values in PAD patients from urban areas contribute to an increased cardiovascular risk in these subjects, who are also exposed to higher stress compared to those living in rural areas [22].

The mutations in the MTHFR gene probably developed during evolutionary processes because of adaptation to the high folic acid intake during the Neolithic period [23]. Heterozygous C677T mutation leads to around 65% activity of the MTHFR enzyme, while the homozygous mutation results in 30% enzyme activity; thus, homozygous mutation can lead to dangerously high serum homocysteine levels. A mild increase in serum HCy levels is up to 30 μmol/L; values between 30 and 60 μmol/L are considered to be moderately elevated, while concentrations above 60 μmol/L are considered to be severe and dangerous. In this study, the peak serum Hcy level was 44.38 μmol/L in a patient with a homozygous C677T MTHFR gene mutation (the normal range of homocysteinemia was 3–12 μmol/L). Significantly higher mean serum homocysteine levels were observed in patients with severe PAD who were homozygous for the C677T variant of the MTHFR gene than in those without this mutation. It is likely that due to the small number of cases examined, no significant difference could be demonstrated between the average serum Hcy concentration of patients with lower limb arteriopathy carrying the heterozygous C677T mutation and patients with PAD without mutations. The homozygous C677T MTHFR mutation affects approximately 8–20% of the European population. According to Khandanpour et al., the presence of the T allele is associated with higher Hcy levels in PAD patients, but the heterozygous (CT) group might not always show a significant difference on its own compared to the wild-type (CC) group [24]. Other studies revealed similar increases in serum homocysteine levels in PAD patients and the general population with homozygous MTHFR mutation [6,10,25].

The management of complicated PAD cases, especially in the presence of gangrene, requires an interdisciplinary approach aimed at minimizing the extent of the amputation in order to improve the patients’ quality of life. Avoiding sepsis is very important, especially in cases where the affected limb is infected with multidrug-resistant bacteria [26]. Wet gangrene generally has a worse prognosis than dry gangrene. The most common bacteria involved are aerobic organisms (*Klebsiella*, *Staphylococcus*, *Streptococcus* strains, etc.), or aerotolerant anaerobic microorganisms like *Clostridium perfringens* [27]. In the present study, aerobic bacteria were found in patients with wet gangrene, some of which were facultative anaerobic bacteria, such as *Escherichia coli*, *Enterobacter cloacae*, or *Leclercia adecarboxylata*. The presence of infections did not exert a significant influence on the values of the blood-count-derived biomarkers assessed in this research.

The pattern of antibiotic resistance was broadly consistent with that observed locally in Romania, although the prevalence of cephalosporin and fluoroquinolone resistance was slightly lower in this study than in the literature. In terms of the prevalence of multiple-drug resistance in Gram-negative bacteria cultured from pus, we observed slightly higher values during the COVID-19 pandemic than the local data reported in the literature [28,29].

The ROC analyses demonstrate that the tested hematological ratios (NLR, PLR, LMR, PMPV) have limited-to-poor diagnostic utility across all tested conditions, including infection, diabetes mellitus, and various mutation statuses. While some parameters occasionally exhibited high sensitivity or specificity for certain conditions (e.g., LMR and NLR for heterozygous mutations), this often came at the cost of the other metric, making them unreliable as standalone markers.

The originality of the study is derived from the assessment of a complex scale of laboratory tests, including biochemical, hematological, microbiological, and genetic testing in a group of PAD patients with and without diabetes mellitus. The prognostic role of certain blood-count-derived biomarkers was demonstrated, which is not commonly used in clinical practice. The corroboration of homocysteinemia with the presence of the most common MTHFR gene mutation increases the complexity of this research. This work represents one of the few studies on the thrombophilia profile in patients with arterial thrombosis, while the vast majority of similar contributions are focused on venous thrombosis.

The limitations of the study are due to the lack of certain data; for example, serum vitamin B_12_ and folic acid levels were not assessed. Smoking habits were not mentioned in all cases, so this important risk factor could not be taken into consideration for the entire patient group; thus, it was not included on the list of surveyed data. Another limitation is that only one mutation (the most common one) was assessed for the MTHFR gene and further tests on the thrombophilia profile were not performed in the selected group of severe cases.

## 5. Conclusions

The correlations found between biomarkers derived from the complete blood count could help clinicians identify cases that are prone to complications and require longer hospital treatment, which require more complex management. Based on consistently low AUC values, none of these parameters alone are recommended for clinical diagnostic purposes in these specific cases, except after further validation or combination with other biomarkers.

In cases where all blood-derived inflammatory markers were within the normal range, the length of hospital stay was significantly shorter.

The LMR value showed a negative correlation with the length of hospital stay.

The outcome of the study emphasized the relationship between elevated serum homocysteine levels and homozygous MTHFR mutation, but no significant difference was observed in the homocysteinemia of heterozygous subjects compared to the wild-type group.

## Figures and Tables

**Table 1:** Proteinemia and albuminemia of PAD patients with and without T2DM before surgery.

Parameter	Mean ± SD	Unit	Normal Range
Proteinemia in PAD patients	6.70 ± 0.96	g/dL	6–8
Albuminemia in PAD patients	3.09 ± 0.55	g/dL	3.5–5.2
**Subsets according to T2DM**			
**Parameter**	**Mean ± SD**	**Unit**	***p*** **Value**
Proteinemia in T2DM PAD patients	6.61 ± 0.8	g/dL	0.5565
Proteinemia in non-T2DM PAD patients	6.77 ± 1.06	g/dL	
Albuminemia in T2DM PAD patients	2.86 ± 0.34	g/dL	0.2478
Albuminemia in non-T2DM PAD patients	3.25 ± 0.63	g/dL	

**Table 2:** ROC analysis for different blood-derived parameters.

Parameter/Clinical Status		Discrimination	AUC	Optimal Cut-Off	Sensitivity	Specificity
PMPV	Infected/non-infected	poor	0.644	40.89	64.7%	67.2%.
	Diabetic/non-diabetic	poor	0.537	39.48	54.9%	57.44%
	Homozygous	poor	0.633	39.07	75.0%	60.0%
	Heterozygous	none	0.486	39.52	83.0%	50.0%
PLR	Infected/non-infected	poor	0.570	133.54	88.2%	35.9%
	Diabetic/non-diabetic	poor	0.563	136.35	78.4%	36.1%
	Homozygous	poor	0.517	307.92	100%	40%
	Heterozygous	poor	0.611	277.16	100%	33.3%
LMR	Infected/non-infected	poor	0.554	645.26	38.2%	75.0%
		poor	0.504	5.63	17.6%	93.8%
	Diabetic/non-diabetic	poor	0.617	2.95	56.9%	63.8%
	Homozygous	poor	0.550	3.03	58.3%	80%
	Heterozygous	poor	0.660	3.76	94.1%	41.7%.
NLR	Infected/non-infected	none	0.483	3.90	55.8%	51.5%
	Diabetic/non-diabetic	poor	0.569	4.07	54.9%	61.7%
	Homozygous	poor	0.617	2.53	75.0%	60.0%
	Heterozygous	poor	0.639	2.85	41.7%	100%

**Table 3:** Type of bacterial pathogens found in the pus of infectious gangrene.

Bacterial Strain	Number of Cases	Remarks
*Staphylococcus aureus*	12	MRSA in 5 cases
*Streptococcus*	7	Mostly group B *(S. agalactiae)*
*Pseudomonas aeruginosa*	5	-
*Enterococcus* spp.	5	-
*Proteus vulgaris*	4	-
*Proteus mirabilis*	4	-
*Escherichia coli*	3	One ESBL-producing strain
*Morganella morganii*	3	-
*Enterobacter cloacae*	3	-
*Serratia marcescens*	2	-
*Citrobacter koseri*	2	-
*Stenotrophomonas maltophilia*	2	-
*Klebsiella oxytoca*	1	-
*Klebsiella pneumoniae*	1	-
*Leclercia adecarboxylata*	1	-

MRSA: methicillin-resistant *Staphylococcus aureus* and ESBL: extended-spectrum beta-lactamase.

## Data Availability

Data are available upon request from the corresponding author.

## Abbreviations

The following abbreviations are used in this manuscript:

A	adenine
Ala	alanine
APTT	activated partial thromboplastin time
C	cytosine
CBC	complete blood count
COVID-19	Coronavirus Disease of 2019
CRP	C-reactive protein
DNA	deoxyribonucleic acid
EDTA	ethylenediaminetetraacetic acid
ESBL	extended spectrum beta-lactamase
G	guanine
Hb	hemoglobin
HCy	homocysteine
INR	international normalized ratio
IQR	interquartile range
LMR	lymphocyte-to-monocyte ratio
MPhARC	Medical and Pharmaceutical Advanced Research Center
MPV	mean platelet volume
MRSA	methicillin-resistant *Staphylococcus aureus*
MTHFR	methylenetetrahydrofolate reductase
NLR	neutrophil-to-lymphocyte ratio
NO	nitric oxide
PAD	peripheral artery disease
PC	platelet count
PCR	polymerase chain reaction
PLR	platelet-to-lymphocyte ratio
PMPV	platelet-to-mean platelet volume ratio
PMR	platelet-to-monocyte-ratio
PT	prothrombin time
ROC	receiver operating characteristic
SD	standard deviation
T	thymine
T2DM	type 2 diabetes mellitus
Val	valine
